# Physical, Social, and Political Inequities Constraining Girls’ Menstrual Management at Schools in Informal Settlements of Nairobi, Kenya

**DOI:** 10.1007/s11524-017-0189-3

**Published:** 2017-09-05

**Authors:** Candace Girod, Anna Ellis, Karen L. Andes, Matthew C. Freeman, Bethany A. Caruso

**Affiliations:** 1 Department of Environmental Health, Rollins School of Public Health, Emory University, Atlanta, GA USA; 2 Department of Global Health, Rollins School of Public Health, Emory University, Atlanta, GA USA

**Keywords:** School, Menstruation, Puberty, Sanitation, Religion, Equity

## Abstract

Access to adequate water and sanitation is limited in informal settlements, contributing to girls’ challenges managing menstruation at school, especially when they cannot access materials to absorb menstrual blood and appropriate facilities for hygiene. This study documents differences between girls’ experience of menstruation at public schools (where the Kenyan government provides menstrual pads) and private schools (where pads are not provided) in two informal settlements of Nairobi, Kenya. Results showed that supply chains to public schools were not reliable, and equitable pad provision was not assured. Girls in private schools struggled to access pads because they were not provided. Sanitation facilities were physically available, but Muslim girls were unable to practice ablution due to the design of toilets in our study schools. Girls experienced fear and anxiety due to harassment from male peers and had incomplete information about menstruation from teachers. Findings suggest that practitioners and policy-makers should acknowledge the diversity of school populations and monitor programs to ensure efforts do not contribute to inequity.

## Introduction

When girls can manage menstruation at school in supportive environments, they report that they are less likely to be absent and can concentrate better in class, potentially improving their educational experience [1–4]. Schools that provide puberty and menstruation education as well as access to water, sanitation, and hygiene (WASH) facilities contribute to an enabling environment that supports girls to attend and participate. These conditions are lacking for many girls in low-income settings [5–7]. Girls are often uninformed and unsure about what is happening to their bodies when they reach menarche, causing fear and confusion [8, 9]. They may not have adequate facilities at school such as safe, private, and clean toilets equipped with functional door locks, water for personal hygiene, or disposal facilities [5, 6, 10–12]. Evidence suggests that women’s education is associated with improvements in economic productivity, decision-making ability, contraception use, age at first birth, family size, maternal mortality and morbidity, and educational attainment among their children [13–18]. Inadequate menstrual hygiene management (MHM) in school may jeopardize girls’ ability to stay in school and achieve better life outcomes for themselves and their future families.

Studies show that poor conditions of WASH facilities contribute to girls’ challenges managing menstruation in Kenya [3, 5]. In 2003, the Kenyan government instituted Free Primary School Education, which increased school enrollment by 13% within the first year, [19, 20] straining existing WASH facilities [21]. While educational opportunities for girls have improved, school systems have not achieved gender parity, and girls have a higher risk of being unenrolled [22, 23]. In an attempt to cater to girls’ needs, the Kenyan government initiated a school health strategy, which includes distributing sanitary pads for girls to administrators in public primary schools, improving the pupil to toilet ratio, and creating disposal mechanisms for pads at public primary and secondary schools [24]. The technical guidelines for national school WASH specify that each toilet block should have ablution facilities, disposal facilities, at least one water point, the girl-pupil to toilet ratio should be 1:25, and boys’ and girls’ toilets should be separated [25]. However, the national average pupil to toilet ratio for girls in primary school was 1:45 in 2013, far from the guideline target [26]. The pupil to water point ratio and the number of children who must share a water point are unknown. Little is known about the specific impact these policies have on girls’ ability to manage their menstruation in school, and whether or not these policies contribute to inequitable experiences among girls in public schools, who benefit from these policies, and girls in private schools, who do not.

Evidence suggests that girls’ experiences of menstruation in informal settlements of Nairobi are worse than the rest of the city [27]. Recent research found that schools in these communities have poor sanitation, impeding girls’ school attendance because they are unable to manage menstruation adequately [28]. Sustainable Development Goal (SDG) 6.2 is to “achieve access to adequate and equitable sanitation and hygiene for all…paying special attention to the needs of women and girls and those in vulnerable situations.” [29] It is currently unknown if girls have equitable sanitation and hygiene in schools of informal settlements when managing their periods. The purpose of this study was to understand if girls in informal settlements of Nairobi, Kenya experience inequity in their physical and social environment based on the type of school they attend (public vs private), the type of WASH facilities available to them, and their religion (Christian or Muslim).

## Methods

### Study Setting

This study took place at schools in the Mukuru and Mathare informal settlements (May–August 2015). Mukuru has approximately 500,000 residents and lies 10 km southeast of Nairobi’s city center. Mathare is 4 km northeast of Nairobi’s city center and has a population of approximately 700,000 [30]. Political and social factors created household water and sanitation characteristics that are far worse in Nairobi’s informal settlements compared to the city at an aggregated level [31]. Only 28% of informal settlement households have water piped into their residence compared to 78% of residents in Nairobi collectively. A greater proportion of informal settlement households need to get water from public taps (20%) or purchase their water from private companies (11%) compared to 14 and 3% of Nairobi residents, respectively [32]. Ninety four percent of informal settlement residents have access to a toilet, but as many as 83% rely on paid public toilets, which forces many residents to use flying toilets (when one defecates into a plastic bag and throws it away) or open defecation [28, 31, 32]. Using these public toilets costs 3–7% of a family’s income, they tend to be over 50 m away from their homes, and every time they walk to the toilet, women risk sexual violence by community members [31].

This research was conducted in partnership with Sanergy, a for-profit sanitation social enterprise operating in Nairobi since 2011. Sanergy constructed modular toilets called “Fresh Life Toilets” (FLTs) in Mukuru and Mathare schools as well as other public spaces throughout Mukuru. A Fresh Life Toilet is a 3 × 5 foot stall that has a squat plate on the floor with two holes leading to separate containers for urine and feces. The waste is collected and transferred to an offsite location daily. Mukuru and Mathare are located in low-lying areas with high water tables, making them prone to flooding. Most toilets in these communities are pit latrines, and whenever flooding occurs, excreta from the pit latrines can contaminate household water sources, which can cause illness. Fresh Life Toilets are specifically designed to prevent this problem by utilizing containers for excreta that are collected and transferred to an offsite location daily.

### School Selection

We purposively selected six Sanergy-affiliated primary schools (grades K-8), based on dominant religion, toilet type, and public or private status. Given the lack of information about the varied needs of Christian and Muslim girls in this context, we chose three schools with a Christian majority and three schools with a Muslim majority. To understand students’ experiences using different toilet designs, we selected three schools with FLTs and three schools with pit latrines/flush toilets. We included a mixture of public and private schools to learn how girls’ experiences may differ if they had government supported access to pads.

### Research Activities

At each school, we conducted one focus group discussion (FGD) with girls, one key informant interview (KII) with the Head Teacher, one school facility observation, and an anonymous question session (AQS). The FGDs, KIIs, and observation tools were adapted from UNICEF’s “WASH in Schools Empowers Girls” Education: Tools for Assessing Menstrual Hygiene Management in Schools [33].

The FGDs, which included 6–11 post-menarcheal girls in grades 6–8, used participatory activities to explore girls’ challenges at school related to menstruation and to identify girls’ perceptions of important toilet characteristics for menstruating girls. Participatory activities were embedded in the FGDs because they have been shown to enable girls to discuss menstruation practices at school more openly than direct questioning alone [34, 35]. In the first FGD activity, “The Ideal Place to Manage Your Period,” participants worked together to draw pictures of the perfect facility for menstrual management and discussed their drawings. In the second activity, “Imagining the Life of a Normal Girl,” participants discussed a hypothetical situation about a peer menstruating at school, how she used the school’s WASH facilities, how she accessed money to buy pads, and her comfort with talking to teachers and peers about menstruation. School administrators selected focus group participants.

KIIs examined how teachers provide menstrual hygiene education, sought their perspective of student behavior with regard to menstrual hygiene, and gathered information about operation of the school water and sanitation facilities.

We observed facilities to document the adequacy of toilet facilities for menstrual management by assessing cleanliness, function, lighting, and privacy. The key informant or the school groundskeeper observed the facilities along with the research team.

The purpose of the AQS was to understand and document girls’ uncertainties about menstruation and answer their questions. This activity was informed by an approach used with girls in Tanzania [10]. All female students in grade 6–8 were allowed to participate, regardless of their menarcheal status. Researchers distributed paper for writing menstruation-related questions anonymously, then collected the questions and provided answers.

We conducted all research activities in English and used the iPhone Voice Memos app (Apple Inc., Cupertino, California) to record FGDs, KIIs, and AQSs. We transcribed audio recordings of interviews and FGDs and consulted Sanergy staff for translating comments made in Sheng (a Swahili-based slang).

### Analysis

All KII and FGD transcripts and typed anonymous questions were analyzed in MAXQDA (version 11, VERBI Software, Berlin). Data analysis began by reading transcripts and writing memos to identify themes. We defined codes to represent those themes, refined them, and applied them to the data. Next, we developed case studies for each school that compared details from each activity. Crafting case studies facilitated data triangulation and an understanding of each school’s unique environment. We subsequently conducted a thematic analysis by retrieving the text by theme and creating tables categorizing the main ideas in each coded segment. Next, we transferred segments to MAXMAPS, a “virtual whiteboard” in MAXQDA, and created idea webs based on common themes, noting the associations and overlaps. Finally, the maps for each theme were synthesized into narrative form, adding nuance and depth to the associations.

### Ethics

Emory University’s Institutional Review Board deemed this research exempt. Approval for work with Sanergy was granted by the Ethical Review Board of Great Lakes University of Kisumu, Kenya. Head Teachers in each school signed in loco parentis consent forms for student participation, and all students and adults provided verbal informed assent/consent before each research activity.

## Results

### School and Participant Information

Public schools were larger than private schools with fairly even numbers of girls and boys (See Table 1). Private schools were located within the informal settlements and served students from that community, while public schools were located 2 km or less from informal settlements and served students who lived in informal settlements as well as those who did not. Almost all of the Muslim students in this study attended public schools. In two of the three schools with FLTs, only girls in grade 6–8 were permitted to use them. All of the public schools had pour flush toilets, and two of the public schools had Fresh Life Toilets in addition to their pour flush toilets. Private schools had only one type of toilet, either fresh life, pour flush, or pit latrine.

**Table 1:** Descriptive statistics for schools (6) studied in Mukuru and Mathare

	Public	Private
	Mean or number (Range or %)	Mean or number (Range or %)
Total number of schools	3 (50%)	3 (50%)
Mukuru	1 (33%)	2 (66%)
Mathare	2 (67%)	1 (33%)
Population	976 (902–1054)	232 (115–295)
Girls	508 (468–560)	119 (69–147)
Primary religion^a^		
Muslim	2	0
Christian	0	3
Muslim and Christian	1	0
Water sanitation and hygiene facilities		
Pupil to functional latrine ratio (girls)^b^	27:1 (24:1–28:1)	88:1 (47:1–147:1)
Pupil to total latrine ratio (girls)	23:1 (21:1–28:1)	88:1 (47:1–147:1)
Pupil to total water points	58:1 (47:1–75:1)	168:1 (97:1–295:1)
Pupil to functional water points ratio, functional (total)	509:1(97:1–902:1)	232:1(115:1–295:1)
Toilet paper present at LEAST sometimes	3	2
Private dustbin in at LEAST 1 latrine stall	3	1
Toilets types		
Pour flush	3	1
Fresh Life Toilets	2	1
Pit latrines	0	1
Locks on any doors	3	2
Formalized pad provision from school	3	0
Extra uniforms provided by school after menstrual accident	1	0

^a^Based on reports from key informants

^b^Functional latrines are those that students were able to use to urinate or defecate, though students were not always able to flush toilets themselves because they did not have access to containers to pour water into the toilet bowl

### Challenges Posed by Policy, School Infrastructure, and Social Norms

Challenges related to WASH and menstrual management varied based on whether pupils attended a public or private school, girls’ socio-economic status, and their religion. All girls lacked access to complete and accurate information about menstruation, and they faced harassment and risk of sexual assault.

#### Access to Pads and WASH Infrastructure Based on School Enrollment

There is a broad array of menstrual materials available in Nairobi, including menstrual cups, tampons, disposable sanitary pads, and reusable pads. However, availability of products is variable. Menstrual pads were the most prevalent menstrual material among the women and girls in this study. While many non-governmental organizations provide girls with reusable sanitary pads, including girls at one of the private schools in this study, disposable pads are still far more popular and convenient for girls at school. Girls in this study said that they use disposable menstrual pads, and in an emergency, they use toilet paper if it is available. None of the participants in this study said that they use reusable pads at school. Any mention of pads in this article refers to disposable sanitary pads. Girls in public schools had greater access to pads and WASH facilities. Access largely determined whether girls stayed in school all day during menstruation and how comfortable they felt managing menstruation at school.

#### The Sanitary Towels Programme Provides Pad Access, but Not for All Girls

Only girls in public schools could expect to receive pads from their teachers when they asked. Public schools were part of the Sanitary Towels Programme sponsored by the Kenyan government. The program, focused on schools in arid parts of the country and in informal settlements, has two primary components: (1) provision of one package of disposable menstrual pads per menstruating girl per month (10 pads, the amount that women and girls typically use during their period), and training for teachers so that they can discuss menstruation with their students appropriately and accurately. When a teacher attends the government sponsored training, their school is eligible to receive menstrual pads for all of the menstruating girls in their school. At private schools, where the program did not apply, girls more frequently reported menstrual stains and missed school than girls in public schools because they lacked money to purchase menstrual pads. While some families could afford to provide disposable pads, girls in every private school discussed their inability to purchase pads as often as they needed. Participants said that non-governmental organizations sometimes donated pads, but the timing was unpredictable, and the quantity was only sufficient for 1 month or less. Consequently, girls in private schools more often expressed fear and anxiety about staining their clothes.

*If she [the teacher] sees you, that you have messed bad, she will tell you to go home and shower and wear a pad…[but] you could go home and find no one in the house and then you cannot come back to school because of the sanitary pads. So there are some lessons that you could miss that day.* ***(FGD, School 1, Private)***

Girls had inequitable access to pads even in public schools where they were provided by the government because not all girls felt comfortable requesting them. Additionally, supplies were not consistently available, and no standards existed to identify girls in greatest need. Participants in focus groups said that they were able to access pads, but that some of their peers lacked the confidence to ask their teacher for pads. Public school teachers described rationing the pads by only giving girls half of the number they might typically need because they did not have enough to give to each girl for the duration of her period. They reported that it had been almost a year since the last pad disbursement, and they were not sure when or if the government would distribute more pads. They did not have an objective way to identify needy students, so they distributed based on their knowledge, potentially overlooking some girls in need.

*Now, we are fearing to give them a whole packet. Even now if they come, I give them maybe 4. And those ones who are coming from the slums, they are the ones I give a whole packet. Because those ones, will stay home until the period is over.* ***(Teacher, School 5, Public)***

#### Access to school WASH facilities improves girls’ ability to manage menstruation

Girls in five of the six schools had limited access to WASH facilities they needed. The public schools we visited had one water point per toilet block as per the national standards [33]; however, the pupil to water point ratio was over twice the ratio in private schools compared to public schools (509:1 public, 232:1 private). The pupil to water point ratio contributed to long lines for handwashing, and participants in one public school reported bullying from older girls in line. Only one school had a private space with access to running water because a former student donated sinks. At that specific public school, girls could ask permission to use the teacher’s toilet block, which had running water, so they could wash their stained clothes or body parts. Girls in this school also had access to extra uniforms if their clothes were stained badly. In every other public and private school, girls went home to take care of stains.

High pupil to latrine ratios at private schools were an obstacle for girls (27:1 public, 88:1 private). Girls in every school felt rushed using the facilities to some degree; however, girls in public school, where the latrine ratio was lower, were less concerned than private school girls. Girls in private school said they felt rushed to finish changing their pads, and they sometimes waited until they could go home.

Participants from both public and private schools were concerned about disposal. FLTs have disposal bins within the stall, so girls in FLT schools (2 public, 1 private) were satisfied. The two private schools without FLTs had either a pit latrine or pour flush toilets. At both places, girls said that they kept their used pads in their pockets instead of putting them in the public trash pile because they did not want others to know they were menstruating. This practice came with other worries:

*Since the pad box is not there, if you put the pad in your pocket it may start smelling or it may fall down and you'll get embarrassed and ashamed.* ***(FGD, School 2, Private)***

In traditional toilets at public schools, disposal bins were in one or two toilet stalls. Sometimes girls threw their used pads on the floor if they could not wait for a stall with a disposal bin, scaring younger children who would urinate or defecate on the floor to avoid going near the used pad. At one school, the janitor refused to clean the toilets when girls threw their pads on the floor; his frustration caused fear and stress for girls.

***Girl 1:*** *He is always angry. If he is cleaning and you go there, you'll make him even more angry.*

***Girl 2:*** *Even you can be beaten.*

***Girl 3:*** *If you just go to fetch water to drink it, you can be beaten.* ***(FGD, School 6, Public School)***

This janitor also locked the door after he had finished cleaning, preventing girls from accessing the toilets. When this happened, girls would have to wait to use the toilet until a regularly scheduled break time, which occurred halfway through the morning classes, at lunch time, or halfway through the afternoon classes. Alternatively, girls could find a teacher to ask the janitor to unlock the door; however, this was particularly problematic for girls who were too shy to talk to their teachers.

#### Inequitable Access Based on Religious Practice

Girls experienced inequity when they practiced ablution. The majority of Muslim students in the study attended public schools—only one participant identified as Muslim at the private schools. During focus groups, Muslim girls said that not all classrooms had bottles for girls to carry water for ablution. When they asked teachers to borrow a bottle from another classroom, the teacher sometimes refused. Girls noted that some teachers would say that the classroom did not have bottles, although the bottle was in sight. Girls described feeling angry and frustrated when this happened. Muslim girls in public schools (which had higher pupil to water point ratios in our sample) described frustration with access to water points. They said that lines were often very long. This was particularly troublesome because they needed to get water before and after using the toilet.

Participants referred to the FLTs as the “Christian toilets” because Muslim students were unable to use them. FLT urine containers were too small and girls were not allowed to perform ablution in them. Instead, Muslim girls used older flush toilets with no functional water supply. A janitor flushed the toilets two or three times per day, so they were far dirtier and rarely had private disposal bins or locks on doors. Three stalls out of eight at one public school were designated as “Muslim toilets,” although Muslims made up the majority of the student body. Those toilets had more feces and urine on the ground, creating further inequity.

### Challenges to Managing Menstruation at School for All Girls

#### Girls Do Not Receive Complete Information about Menstruation

Girls at all schools experienced challenges accessing complete and accurate information about menstruation. Teachers at every school said they taught girls about menstruation beginning in grade six or after they reached menarche. Those who reached menarche prior to grade six received no prior information. Girls reported that teachers explained how to wear a sanitary pad and how to clean their bodies during menstruation, coupled with warnings about not getting pregnant and/or not getting raped. Consequently, girls were confused about the relationship between sex, rape, pregnancy, and menstruation.

***Girl 1:*** *[The teacher says] You stop playing with boys on your period.*

***Girl 2:*** *On your period, you can get pregnant.* ***(FGD, School 3, Private)***

*When someone is continuing with the period and is raped is there any chance to get pregnant?*

***(AQS, School 4, Public School)***

Girls in every school had fear and anxiety about getting infections. They worried about negative health outcomes due to poor menstrual management, and they believed that “splash back” (urine splattering onto the vulva) could cause urinary tract infections, gonorrhea, or infertility—regardless of menstrual status. Girls reported that teachers said using anything but a disposable pad would cause these ailments:

*[She said] our private part is just like a mouth. And if we use tissue paper [instead of a sanitary pad]... [It could get into the vagina], and you may suffer with these diseases. [You may get] the disease that cannot be cured--You may not be able to have a kid.* ***(FGD, School 1, Private)***

Participants also said they were less able to concentrate on their school work because of fear and anxiety about menstrual accidents.

#### Many Girls Experience Gender-Based Harassment and Assault

Girls indicated that the onset of menstruation introduced new gendered inequities that inhibited their sense of comfort and safety at school. Harassment and assault from boys as well as the threat of rape from classmates and community members hindered the level of safety girls felt in their school.

***Interviewer:*** *How do boys tease?*

***Girl 1:*** *They may say, now you're a mother.*

***Girl 2:*** *They will say that you will have 15 babies*

***Girl 1:*** *Or they say you are raining [hurtful slang term for menstruation].* ***(FGD, School 5, Public)***

Girls at every school expressed fear and stress related to boys. In one school, girls stopped using the FLTs even though they were far cleaner than the flush toilets without a water supply because boys were trying to peak into the stall through vents in the doors. Girls and teachers said that boys regularly harass girls. Girls in one focus group described multiple occasions in which boys knew that a girl had a blood stain on her uniform; the boys found ways to make the girl stand up to expose the stain.

***Girl 1:*** *They may not let you stay in peace in class...They may take all of [your books] So that you [have to] stand up.*

*[…]*

***Girl 2:*** *You stand up from your [desk] and you don’t know that you have messed [had a menstrual accident].* ***(FGD, School 5, Public)***

Girls in this focus group also talked about trying to play soccer or run around to divert attention from menstruation. Unfortunately, half of the girls did not feel comfortable playing games because boys touched them inappropriately.

***Girl 1:*** *Some boys, they pretend that they're playing with you. Like, playing football. When they're playing, they come, and touch your breast. You think that's it's just a play, but they have...*

***Girl 2:*** *Their intensions*

***Girl 3:*** *We would like it to stop*

***Girl 1:*** *We feel devastated* **(FGD, School 5, Public)**

While this was the only incidence in which girls discussed a specific incidence of sexual assault, girls in four of the six schools asked questions about boys touching them inappropriately or about boys raping them.

Teachers placed the onus on girls to prevent harassment. They said that girls must either ignore boys when they tease (KII, School 1, Private; KII, School 6, Public), or teachers place blame on girls by saying that teasing happens because girls are “mixed up with boys” (KII, School 2, Private).

Some participants disclosed that they had been raped, and they did not feel safe in their communities, while others were anxious about being raped.

*If your father’s friend came and tell you to have sex with him, what would you do?* ***(AQS, School 4, Public)***

Teachers told girls that they needed to be wary of boys and men once they reached menarche and that family members could be dangerous because they might misconstrue friendliness as a sexual advance.

***Girl 1:*** *Even hugging your brothers. You should keep away from them*

***Girl 2:*** *If you're grown up let's say,*

***Girls 3:*** *Even your uncle, say he has just come from Mombasa, do not run to him and hug him.*

***Girl 4:*** *Go and greet him with hands. Say hi with words*

***Girl 1:*** *Don't go and jump*

***Girl 4:*** *You should not be that close to men. Even if your father has come with a male friend to the house, it is not good to go and greet him.* ***(FGD, School 5, Public)***

## Discussion

We found that girls in our study schools were unable to access complete and accurate information about what menstruation is and how to hygienically manage menstruation. Girls faced inequities in access to menstrual pads and WASH facilities necessary to manage menstruation based on the type of school they attended, their level of individual agency, as well as their religion. Girls in all schools had inequitable schooling experiences because of their gender, and many feared sexual harassment or assault from peers at school.

### Inequities Based on Enrollment in Public or Private School

Girls in public school had greater access to the materials and WASH facilities necessary to manage menstruation at school, compared to those in private schools. No other studies about this topic compare public and private schools. Girls at public school less commonly reported having a menstrual accident, staining their clothes, or going home when they were menstruating because their teachers supplied them with sanitary pads and they had better access to toilets, despite having access to fewer water points. The Sanitary Towels Programme only benefits girls in public schools. Similar to other research in Nairobi informal settlements [31], participants in private school reported that they sometimes missed school due to inadequate facilities and menstrual materials. Not requiring private schools to adhere to state policies contributes to inequitable school experiences for girls. Private schools are important in informal settlements because the Kenyan government has failed to create public schools there [36]. Mathare is home to 300,000 school-aged children, but there are only four public schools, serving 4500 children at most [37]. Many development organizations support the promotion of private schools in underserved communities because they operate at cheaper costs and increase access to education [36]. However, none of the private schools in this study met the national standards for school WASH, which is consistent with the majority of private schools in Nairobi informal settlements [38]. Attendance at these schools makes children less likely to progress to secondary school, severely limiting their economic potential as adults [39]. Thus, private schools increase access to education for some students, but they are not equal to public schools, putting children who reside in informal settlements at a greater disadvantage compared to the average Nairobi child [38]. The Alternative Provision of Basic Education and Training (APBET) program, is a pathway for private schools to gain some government funding to pay teachers, purchase classroom materials, and improve facilities if they abide by national school standards such as national WASH guidelines, having space for a playground, teacher training requirements, and tenancy agreements or ownership of the schools’ land. However, it is unclear if small, poor, private schools can meet these standards [40]. APBET will include the Sanitary Towels Programme once fully underway but may take several years to be fully enacted [40]. The Kenyan government should expand the Sanitary Towels Programme to private schools now in order to reach all girls in need.

### Inequities within Public Schools

Public schools in this study benefitted greatly from policies including the Sanitary Towels Programme and WASH guidelines; however problems with implementation and access persisted. Teachers did not receive enough pads to give a supply to each student every month. Some girls were too shy to go to their teachers when they were menstruating, contributing to inequity because girls gained access to resources based on their level of agency, not necessarily their level of need. Teachers were also unsure of when they would receive more pads from the government and had not received a supply in almost a year. As Paulino and Thinguri noted in their study, the Sanitary Towels Programme is an innovative idea, but implementation needs improvement [41]. As called for elsewhere, MHM program delivery mechanisms need to be evaluated to ensure provision of resources for girls are distributed effectively [42]. Further, national governments, and government entities like the Ministry of Education specifically, need to allocate appropriate funds for MHM programs and monitoring of programs to ensure they are inclusive of even the most vulnerable [43]. Moving forward, the Kenyan government should make an effort to publically indicate how often schools should receive pads, who qualifies for them, and how they should be distributed to students so that students, parents, and teachers can be aware of what they are entitled to and can hold actors accountable if the program is not performing as intended.

### Inequity within Schools and the Sustainable Development Goals

Improving sanitation has been identified as a key component to improving health in informal settlements [44, 45]. Improving sanitation facilities increases privacy, dignity and hygiene conditions for menstruating women and girls in informal settlements [31]. Kenya’s national school sanitation guidelines include menstrual management facilities; however, inconsistencies and inequities persist regarding access to toilets, water, disposal, ablution spaces, and pads. The schools in this study had toilets available and a large number of pupils used them. However, a toilet’s presence at school, and use by large numbers of students, does not ensure equity in access [46, 47]. Several factors limited girls’ access to toilets even when there appeared to be enough available. Some girls said that they go home when the toilets are too dirty because they do not feel comfortable changing their pads or they feel too rushed to clean a stain from their skirt due to long lines for toilet use. There were similar findings in the Philippines and other parts of Kenya [6, 47, 48].

Religious practices determined whether or not participants could access facilities. Muslim girls had to compromise their religious beliefs or use older, dirtier toilets when the new toilets did not accommodate ablution. Additionally, teachers blocked access to water containers in one of the schools. National WASH guidelines require ablution facilities in schools, but these findings show that establishing national guidelines did not ensure equity. Freeman et al. [49] discuss similar findings in their comparative study of equity of access to WASH in schools in Kyrgyzstan, Malawi, the Philippines, Timor-Leste, Uganda, and Uzbekistan.

Fears about security also prevented access to facilities. Girls did not use toilets if they were afraid of boys sexually harassing them while changing pads, an issue also reported in the Philippines [50]. Girls were not comfortable playing with their male peers because of fear that they would be touched inappropriately. Teachers absolved boys of their behavior toward girls, saying girls should not be “mixing up” with boys, solidifying the idea that boys are not accountable for their actions [51, 52]. When teachers told girls that they must be aware of men and other boys now that they are menstruating, they implied that the girls are now sexual objects [3, 53, 54]. This discourse may lead girls to believe that they are at fault when they are harassed or sexually assaulted, which is harmful to their mental health, and may be a factor influencing girls’ lower belief in a just world compared to boys [51, 55]. This work provides further evidence that gender parity in the classroom does not amount to gender equity in school [56, 57].

The factors outlined above undermine the UN’s goal of equitably meeting WASH targets [46]. Research guidance recommends an expanded set of questions for monitoring the availability of menstruation-relevant facility characteristics [42]. However, under current Joint Monitoring Program (JMP) data collection procedures, informal settlements are not considered to be official data collection sites. The JMP has medium/long-term plans to include informal settlements in its data collection, but there is no clear indication of when that may be [46]. Few other options exist for collecting data in informal settlements, and policy-makers rarely have what they need to make informed decisions [58]. Therefore, the poorest segments of the urban population with the most under-resourced schools will be overlooked. Targeting data collection to include school populations that may be otherwise neglected is necessary to create a more accurate depiction of WASH in school settings.

### Menstrual Hygiene and Reproductive Health

Teachers in this study discussed menstruation, sexuality, and pregnancy together, but they did not present concrete information that could help girls understand their changing bodies and how these topics are related. Studies in Ghana, Bolivia, and Kenya describe mothers and teachers conflating topics similarly [3, 52, 53, 59, 60]. Researchers have called for a multi-sectoral approach to MHM incorporating the education, WASH, and sexual/reproductive health sectors, but movement toward this objective is slow [3, 42, 61, 62]. Providing concrete information about sex, pregnancy, and menstruation can help girls to have more self-confidence and improve their ability to navigate sexual relationships [62]. This is especially important, as girls in informal settlements are more likely to engage in risky sexual behavior [63]. Studies show that provision of menstrual materials reduces incidence of sexually transmitted infections, purportedly by reducing girls’ needs to engage in transactional sex in exchange for menstrual products [64]. Provision of menstrual cups specifically has been shown to reduce the incidence of bacterial vaginosis [64]. Furthermore, teachers and school administrators must work to create school environments where girls feel safe from sexual assault, and emphasize that sexual assault is not the fault of the victim.

### Strengths and Limitations

Our focus on informal settlements and girls from both public and private schools with different types of facilities, and from a variety of economic and religious backgrounds is a strength of this work. Prior research has not investigated equity in access or differences in experience for Muslim and Christian girls in the same WASH setting. Interventions that aim to ameliorate girls’ challenges with menstruation at school focus primarily on education and health outcomes; [65] however, this study suggests that programs should also evaluate equity of intervention experience and uptake to ensure no unintentional harms transpire.

In future research, FGDs with Muslim girls should be held separately. Muslim girls did not seem completely comfortable sharing their opinions about facilities at school when Christian girls were present. Only girls in grades 6–8 participated in focus groups, but teachers mentioned that some girls in lower grades had reached menarche. The inclusion of these younger girls would have provided insight specific to their age set.

## Conclusion

Through qualitative research with girls attending schools in Nairobi informal settlements, we found that girls in private schools had worse experiences of menstruation compared to girls in public schools. Muslim girls in public schools did not have equitable access to the WASH facilities for managing their menstruation adequately. Girls in every school faced harassment from their male peers, and many experienced sexual assault. This study highlights the inequitable experiences girls have when managing menstruation in school. Approaches to MHM should consider equitable access to WASH facilities and materials for all users and education that addresses menstruation in the context of larger pubertal changes.

## References

[1] Montgomery P, Ryus CR, Dolan CS, Dopson S, Scott LM. Sanitary pad interventions for girls’ education in Ghana: a pilot study. *PloS One*. 2012;7(10):e48274. 10.1371/journal.pone.0048274 PMC3485220 23118968

[2] Mason L, Laserson K, Oruko K (2015). Adolescent schoolgirls’ experiences of menstrual cups and pads in rural western Kenya: a qualitative study. Waterlines.

[3] McMahon SA, Winch PJ, Caruso BA (2011). ‘The girl with her period is the one to hang her head’Reflections on menstrual management among schoolgirls in rural Kenya. BMC international health and human rights.

[4] Trinies V, Caruso B, Sogoré A, Toubkiss J, Freeman M (2015). Uncovering the challenges to menstrual hygiene management in schools in Mali. Waterlines.

[5] Alexander KT, Oduor C, Nyothach E (2014). Water, sanitation and hygiene conditions in Kenyan rural schools: are schools meeting the needs of menstruating girls?. Water.

[6] Caruso BA, Dreibelbis R, Ogutu EA, Rheingans R (2014). If you build it will they come? Factors influencing rural primary pupils’ urination and defecation practices at school in western Kenya. Journal of Water Sanitation and Hygiene for Development.

[7] Connolly S, Sommer M (2013). Cambodian girls’ recommendations for facilitating menstrual hygiene management in school. Journal of Water Sanitation and Hygiene for Development.

[8] Fatusi AO, Hindin MJ (2010). Adolescents and youth in developing countries: health and development issues in context. J Adolesc.

[9] Ali TS, Rizvi SN (2010). Menstrual knowledge and practices of female adolescents in urban Karachi, Pakistan. J Adolesc.

[10] Sommer M (2010). Putting menstrual hygiene management on to the school water and sanitation agenda. Waterlines.

[11] Garg R, Goyal S, Gupta S (2012). India moves towards menstrual hygiene: subsidized sanitary napkins for rural adolescent girls—issues and challenges. Matern Child Health J.

[12] Crofts T, Fisher J (2012). Menstrual hygiene in Ugandan schools: an investigation of low-cost sanitary pads. Journal of Water Sanitation and Hygiene for Development.

[13] Ainsworth M, Beegle K, Nyamete A (1996). The impact of women’s schooling on fertility and contraceptive use: a study of fourteen sub-Saharan African countries. The World Bank Economic Review.

[14] Martin TC. Women’s education and fertility: results from 26 demographic and health surveys. *Stud Fam Plan*. 1995:187–202. 7482677

[15] McAlister C, Baskett TF (2006). Female education and maternal mortality: a worldwide survey. J Obstet Gynaecol Can.

[16] Hatt LE, Waters HR (2006). Determinants of child morbidity in Latin America: a pooled analysis of interactions between parental education and economic status. Soc Sci Med.

[17] UNFPA. Empowering women, developing society: Female Education in the Middle East and North Africa: Population Reference Bureau. Available at: http://www.prb.org/Publications/Reports/2003/EmpoweringWomenDevelopingSocietyFemaleEducationintheMiddleEastandNorthAfrica.aspx." Date Accessed: August 24, 2017.

[18] Roudi-Fahimi F, Moghadam V. Empowering women, developing society: Female Education in the Middle East and North Africa. Washington, DC: Population Reference Bureau; 2003. p. 1-8.

[19] Njue EK, Muthaa GM (2015). Influence of availability of sanitary facilities on the participation of the girl-child in public primary schools in Garissa County, Kenya. Open Journal of Social Sciences.

[20] Elimu Yetu Coalition. Monitoring of the free primary education and establishing the unit cost of primary education in Kenya. Nairobi, Kenya; 2004.

[21] UNESCO. Challenges of implementing free primary education in Kenya. Available at: http://unesdoc.unesco.org/images/0015/001516/151654eo.pdf. Accessed 30 Sept 2016.

[22] UNESCO. UNESCO global partnership for girls’ and women’s education-one year on: Kenya. Available at: http://www.unesco.org/eri/cp/factsheets_ed/KE_EDFactSheet.pdf. Date Accessed: August 24, 2017.

[23] King N, Dewey C, Borish D (2015). Determinants of primary school non-enrollment and absenteeism: results from a retrospective, convergent mixed methods, cohort study in rural western Kenya. PLoS One.

[24] Sharif S, Godia G, Wamae A, Migiro S, Muriithi A, Kabaka S. National school health strategy implementation plan 2010–2015. Nairobi, Kenya: Ministry of Public Health and Sanitation & Ministry of Education; 2010.

[25] Kenya Ministry of Education M. Primary school design. Nairobi, Kenya: School Infrasturcture Management Unit, SIMU; 2010.

[26] NTA. School report card 2013. Nairobi, Kenya: DFID. p. 2014.

[27] Crichton J, Okal J, Kabiru CW, Zulu EM (2013). Emotional and psychosocial aspects of menstrual poverty in resource-poor settings: a qualitative study of the experiences of adolescent girls in an informal settlement in Nairobi. Health care for women international.

[28] Corburn J, Hildebrand C (2015). Slum sanitation and the social determinants of women’s health in Nairobi, Kenya. J Environ Public Health.

[29] United Nations Economic and Social Council. Report of the inter-agency and expert group on sustainable development goal indicators. New York, NY; 2016.

[30] Darkley D, Kariuki A (2013). A study on quality of life in Mathare, Nairobi, Kenya. J Hum Ecol.

[31] Corburn J, Karanja I. Informal settlements and a relational view of health in Nairobi, Kenya: sanitation, gender and dignity. *Health Promotion International*. 2014;31(2):258-269. 10.1093/heapro/dau100 25421267

[32] APHRC. Population and health dynamics in Nairobi ’ s informal settlements: report of the Nairobi cross-sectional slums survey 2012. Nairobi, Kenya: African Population and Health Research Center; 2014.

[33] Caruso BA, Long JL, Yerian SE (2013). WASH in schools empowers girls’ education: tools for assessing menstrual hygiene management in schools.

[34] Long J, Caruso B, Freeman M, Mamani M, Camacho G, Vancraeynest K (2015). Developing games as a qualitative method for researching menstrual hygiene management in rural Bolivia. Waterlines.

[35] Sommer M (2009). Ideologies of sexuality, menstruation and risk: girls’ experiences of puberty and schooling in northern Tanzania. Culture, health & sexuality.

[36] Oketch M, Ngware M (2010). Free primary education still excludes the poorest of the poor in urban Kenya. Dev Pract.

[37] Economist. The $1-a-week school. Available at: http://www.economist.com/news/leaders/21660113-private-schools-are-booming-poor-countries-governments-should-either-help-them-or-get-out. Accessed 15 August 2015.

[38] Edwards DB, Klees SJ, Wildish J. Dynamics of low-fee private schools in Kenya: governmental legitimation, school-community dependence, and resource uncertainty: Philadelphia, PA: Drexel University; 2015.

[39] Zulu EM, Beguy D, Ezeh AC (2011). Overview of migration, poverty and health dynamics in Nairobi City’s slum settlements. Journal of Urban Health.

[40] Education KMo Registration guidelines for Alternative Provision of Basic Education and Training (APBET) Available at: https://www.concern.net/sites/default/files/media/resource/alternative_provision_of_basic_education_and_training_apbet_option_2_cover.pdf. Accessed 12 Oct 2016.

[41] Paulino S, Thinguri R. Critical analysis of the effectiveness of the government policy on provision of sanitary pads to the needy girls in public primary schools in Kenya. *Researchjournali’s Journal of Education*. 2015;3(6):1-8.

[42] Phillips-Howard PA, Caruso B, Torondel B, Zulaika G, Sahin M, Sommer M. Menstrual hygiene management among adolescent schoolgirls in low-and middle-income countries: research priorities. *Glob Health Action*. 2016;9 10.3402/gha.v9.33032 PMC5148805 27938648

[43] Sommer M, Caruso BA, Sahin M (2016). A time for global action: addressing girls’ menstrual hygiene management needs in schools. PLoS Med.

[44] Ezeh A, Oyebode O, Satterthwaite D, et al. The history, geography, and sociology of slums and the health problems of people who live in slums. Lancet. 2016; 10.1016/S0140-6736(16)31650-6 27760703

[45] Kimani-Murage EW, Ngindu AM (2007). Quality of water the slum dwellers use: the case of a Kenyan slum. Journal of Urban Health..

[46] JMP. WASH post-2015 proposed indicators for drinking water, sanitation and hygiene. http://www.who.int/water_sanitation_health/monitoring/coverage/wash-post-2015-rev.pdf?ua=1/: World Health Organization, UNICEF, Joint Monitoring Program; 2015. Date Accessed: August 24, 2017.

[47] Ellis A, Haver J, Villasenor J, et al. WASH challenges to girls’ menstrual hygiene management in Metro Manila, Masbate, and South Central Mindanao, Philippines. *Waterlines.* 2016;[In Press].

[48] Nyothach E, Alexander KT, Oduor C (2015). Handwashing for menstrual hygiene management among primary schoolgirls in rural western Kenya. Waterlines.

[49] Freeman M, Erhard L, Fehr A, Ogden S. Equity of access to WASH in schools: a comparative study of policy and service delivery in Kyrgyzstan, Malawi, the Philippines, Timor-Leste, Uganda and Uzbekistan: UNICEF, New York, NY.; 2011.

[50] Haver J, Caruso BA, Ellis A (2012). WASH in schools empowers Girls’ education in Masbate province and metro manila, Philippines.

[51] Ruto SJ (2009). Sexual abuse of school age children: evidence from Kenya. J Int Coop Educ.

[52] Jewitt S, Ryley H (2014). It’s a girl thing: menstruation, school attendance, spatial mobility and wider gender inequalities in Kenya. Geoforum.

[53] Joshi D, Buit G, González-Botero D, Sahin M (2015). Menstrual hygiene management: education and empowerment for girls?. Waterlines.

[54] Mason L, Nyothach E, Alexander K (2013). ‘We keep it secret so no one should know’—a qualitative study to explore young schoolgirls attitudes and experiences with menstruation in rural western Kenya. PLoS One.

[55] Tavrow P, Withers M, Obbuyi A, Omollo V, Wu E. Rape myth attitudes in rural Kenya: toward the development of a culturally relevant attitude scale and “blame index”. Journal of interpersonal violence. 2013;0886260512471086 10.1177/0886260512471086 23300194

[56] Thomas MA, Rugambwa A. Gendered aspects of classroom practice. *Teaching in Tension*: Springer; 2013:133–148.

[57] Sommer M (2010). Where the education system and women’s bodies collide: the social and health impact of girls’ experiences of menstruation and schooling in Tanzania. J Adolesc.

[58] Elsey H, Thomson DR, Lin R, Maharjan U, Agarwal S, Newell J (2016). Addressing inequities in urban health: do decision-makers have the data they need? Report from the urban health data special session at international conference on urban health Dhaka 2015. Journal of Urban Health..

[59] Crichton J, Ibisomi L, Gyimah SO (2012). Mother-daughter communication about sexual maturation, abstinence and unintended pregnancy: experiences from an informal settlement in Nairobi, Kenya. J Adolesc.

[60] Long J, Caruso BA, Lopez D (2013). WASH in schools empowers girls’ education in rural Cochabamba, Bolivia.

[61] Kirk J, Sommer M. Menstruation and body awareness: linking girls’ health with girls’ education. Royal Tropical Institute (KIT), Special on Gender and Health. 2006:1–22. http://www.susana.org/_resources/documents/default/2-1200-kirk-2006-menstruation-kit-paper.pdf. Accessed July 20, 2017.

[62] Sommer M, Sutherland C, Chandra-Mouli V (2015). Putting menarche and girls into the global population health agenda. Reprod Health.

[63] Kabiru CW, Beguy D, Undie C-C, Zulu EM, Ezeh AC (2010). Transition into first sex among adolescents in slum and non-slum communities in Nairobi, Kenya. J Youth Stud.

[64] Phillips-Howard PA, Nyothach E, ter Kuile FO (2016). Menstrual cups and sanitary pads to reduce school attrition, and sexually transmitted and reproductive tract infections: a cluster randomised controlled feasibility study in rural western Kenya. BMJ Open.

[65] Hennegan J, Montgomery P (2016). Do menstrual hygiene management interventions improve education and psychosocial outcomes for women and girls in low and middle income countries? A systematic review. PLoS One.

